# 

**Published:** 1845-06

**Authors:** 


CONTENTS:

Cases of Surgery. By DANIEL BRAINARD, M. D., Prof. of Surgery in Rush Medical College, 33
Letter from Dr. Blaney, - 35

BIBLIOGRAPHICAL NOTICES.

The Principles of Surgery. By JOHN MILLER, Professor of Surgery in the University of Edinburgh, &c., &c. - - 37
The Missouri Medical and Surgical Journal. May, 1845. - 39

PRACTICAL MEDICINE, &c.

Dr. Buck on the Use and Abuse of Medicine, - - - 39
On the Use of Large Doses of Quinine.—Report of a Commitee of the Medical Department of the National Institute, on Dr. Buck's Paper "On the Use and Abuse of Medicine," - - 43
Rush Medical College, - 48
To Readers, Correspondents, &c. 48

